# Domain motions of Argonaute, the catalytic engine of RNA interference

**DOI:** 10.1186/1471-2105-8-470

**Published:** 2007-11-30

**Authors:** Dengming Ming, Michael E Wall, Kevin Y Sanbonmatsu

**Affiliations:** 1 Computer, Computational, and Statistical Sciences Division, Los Alamos National Laboratory, Los Alamos, USA; 2 Bioscience Division, Los Alamos National Laboratory, Los Alamos, USA; 3 Center for Nonlinear Studies, Los Alamos National Laboratory, Los Alamos, USA; 4 Theoretical Biology and Biophysics Group, Theoretical Division, Los Alamos National Laboratory, Los Alamos, USA

## Abstract

**Background:**

The Argonaute protein is the core component of the RNA-induced silencing complex, playing the central role of cleaving the mRNA target. Visual inspection of static crystal structures already has enabled researchers to suggest conformational changes of Argonaute that might occur during RNA interference. We have taken the next step by performing an all-atom normal mode analysis of the *Pyrococcus furiosus *and *Aquifex aeolicus *Argonaute crystal structures, allowing us to quantitatively assess the feasibility of these conformational changes. To perform the analysis, we begin with the energy-minimized X-ray structures. Normal modes are then calculated using an all-atom molecular mechanics force field.

**Results:**

The analysis reveals low-frequency vibrations that facilitate the accommodation of RNA duplexes – an essential step in target recognition. The *Pyrococcus furiosus *and *Aquifex aeolicus *Argonaute proteins both exhibit low-frequency torsion and hinge motions; however, differences in the overall architecture of the proteins cause the detailed dynamics to be significantly different.

**Conclusion:**

Overall, low-frequency vibrations of Argonaute are consistent with mechanisms within the current reaction cycle model for RNA interference.

## Background

RNA interference, or RNAi, is a conserved mechanism whereby double-stranded RNAs (dsRNAs) silence protein-coding genes that contain sequence fragments complementary to those on the dsRNAs [1-3]. In RNAi, the RNase III family enzyme, called Dicer, cleaves the dsRNA into small interfering RNAs (siRNAs) of ~21 nt [4]. These small RNAs guide the silencing activities within the RNA-induced silencing complex (RISC). Proteins in the Argonaute family are critical to the RISC complex, playing essential roles in substrate selection and mRNA cleavage ([5,6] and references therein).

Argonaute proteins are multi-domain proteins and contain both PAZ and PIWI modules [7]. Structures of PAZ domains complexed with RNA demonstrated that siRNA recognition entails binding of the 3'-end by highly conserved aromatic residues [8,9].

Recently two atomic structures of intact, prokaryotic Argonaute proteins were obtained using X-ray crystallography, with the *Pyrococcus furiosus *(*Pf*-Ago) protein determined to 2.2 Å [10] and the *Aquifex aeolicus *(*Aa*-Ago) protein determined to 2.9 Å [11]. Although the precise role of Argonaute proteins in prokaryotes is currently unknown, these crystal structures have yielded significant insight into mechanisms of eukaryotic Argonaute proteins; in addition, *Aa*-Ago has been shown to be capable of ssDNA- or ssRNA-directed cleavage of RNA [11].

**Figure 1:**
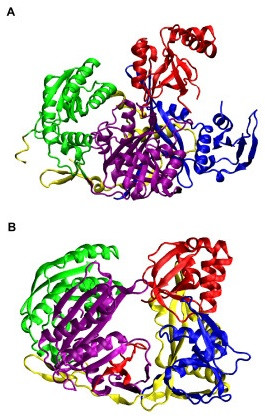
Crystal structures of Argonaute proteins. (a) *P. furiosus *Argonaute (*Pf*-Ago, PDB code 1U04 [10]) adopts crescent-shaped (1+3) architecture with the N-terminal domain (*blue*), the middle domain (*green*), and the PIWI domain (*purple*) as the base and the PAZ domain (*red*) at the top. The linker region is yellow. (b) *Aquifex aeolicus *Argonaute (*Aa*-Ago, PDB code 1YVU [11]) adopts bi-lobal architecture (2+2) with the PAZ and N-terminal domains as one lobe and the PIWI and middle domain as another lobe. The domains of *Aa*-Ago have the same color scheme as that of *Pf*-Ago. This color scheme is used in all subsequent figures. This and all subsequent structural figures were created using VMD [25].

The overall structure of *Pf*-Ago is composed of a 'crescent-shaped' base made up of the N-terminal, Mid- and PIWI- domains, with the PAZ domain mounted above the base by a 'stalk'-like region (Figure 1a) [10]. A DDH motif (D558, D628, H745) with a divalent metal ion Mn^2+ ^has been identified as the mRNA cleavage activity site in the PIWI domain from the *Pf*-Ago-Mn^2+ ^complex [12]. Structure-directed mutagenesis experiments have demonstrated that the PIWI domain of Argonaute is the catalytic unit of the RISC [13]. Later crystal structures of RNA-associated PIWI proteins from diverse species support the similarity of PIWI to RNase H and confirm its mRNA-cleavage-related catalytic function [11,12,14,15].

The structure of the *Aa*-Ago protein is similar to that of the *Pf*-Ago protein in that it has the same domain ordering. However, its overall architecture is better described as a [2+2] bi-lobal protein with PAZ-containing (N-terminal and PAZ domains) and PIWI-containing (Mid and PIWI domains) lobes and the so-called "PIWI box" close to a hinge linking the two lobes (Figure 1b) [11]. In contrast, the *Pf*-Ago protein's architecture has been described as a [1+3] structure with the PAZ domain connected to the N-PIWI-MID crescent base [10].

**Figure 2:**
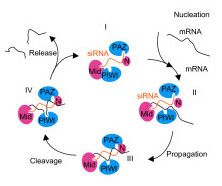
A schematic of the reaction cycle of guide strand-mediated mRNA cleavage procession [11]. Conformer I: single-stranded siRNA binding in the Argonaute as a template strand with its 3' end associated with the binding cleft in PAZ domain. Conformer II: mRNA strand enters and forms Watson-Crick base pair with the template siRNA strand. Conformer III: the 3' end of siRNA strand shifts to bind to N-terminal domain. Base-pairing between the two strands is completed and mRNA strand is ready for cleavage. Conformer IV: the cleaved mRNA strand is loosely paired with template strand and is ready to dissociate from the protein. Transition I to II corresponds to the mRNA nucleation step, transition II to III corresponds to propagation and adjustment, transition III to IV is the cleavage step and transition IV to I is the product release step.

Insights from crystal structures of prokaryotic Argonaute proteins have led to recently proposed models of the RNAi reaction cycle in eukaryotes [6,11,16]. For the purposes of discussion, we refer to this cycle as the Patel Model, which includes guide RNA-directed mRNA loading, cleavage, and product release. In the Patel model, Argonaute assumes four different conformations (Figure 2). Large-scale motions of Argonaute are required to step through the different conformations of the model.

The conformational changes in the Patel Model suggest that large-scale flexibility and domain motions might play a critical role in the RISC machinery. Although the Debye-Waller factors (B-factors) provide insight into the Argonaute's flexibility within the crystalline environment, due to crystal packing constraints, the crystallographic data cannot provide direct observations of the large-scale motions. Therefore, to gain insight into the internal flexibility and accessible conformations of Argonaute, we have used an empirical, molecular-mechanics force field to perform all-atom normal mode analysis (NMA) [17-19] on *Pf*-Ago and *Aa*-Ago. We wished to perform normal-modes analysis using a molecular-mechanics force field instead of an elastic network model [20,21] because we were specifically interested in learning what local and global motions are favored within the context of an all-atom molecular mechanics model that includes explicit terms for electrostatic, van der Waals, and local geometry potentials; the analysis was performed using the CHARMM [31] molecular simulation program (see Methods). The normal modes show hinge motions, torsional motions, and breathing motions which are relevant to specific mechanisms proposed in the Patel model: the biological processes of mRNA/siRNA association and disassociation; and the shift of the 3'end of the guide strand RNA between the PAZ domain and the N/PAZ domain channel.

## Results

### Overall fluctuations

**Figure 3:**
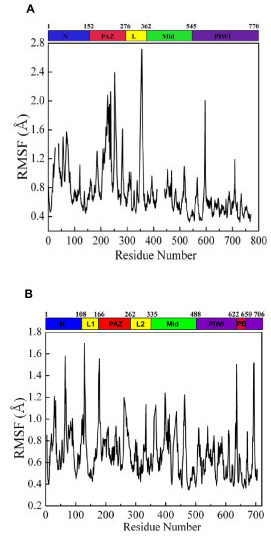
The root-mean-square fluctuations of C_α _atoms at *T *= 300 K calculated using normal mode analysis for (A) *Pf*-Ago and (B) *Aa*-Ago.

**Figure 4:**
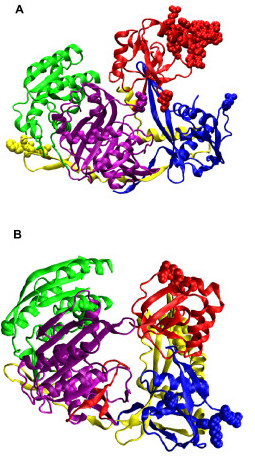
The residues that correspond to several of the highest peaks in Figure 3 as van der Waals spheres for (a) *Pf*-Ago and (b) *Aa*-Ago.

Figure 3 shows the root-mean-squared fluctuations (RMSFs) of C_α _atoms *vs*. residue number for the *Aa*-Ago and the *Pf*-Ago proteins. RMSFs were calculated with Equation (4) at the ambient temperature of *T *= 300 K using the first 500 modes. The correlation between the above RMSFs with that calculated using only the first 15 modes is 0.85 for *Aa*-Ago and 0.93 for *Pf*-Ago, demonstrating that in this case, the low-frequency modes dominate the fluctuation spectrum, as found in normal-mode analyses of other proteins [17-19,22,23]. For *Pf*-Ago, the range of fluctuations is wider in the N-terminal and PAZ domains than in the Mid and PIWI domains. The mean value of C_α_-RMSFs in the N-terminal and PAZ domains is 0.54 Å, which is larger than the mean value of 0.33 Å in the Mid and PIWI domains. By contrast, for *Aa*-Ago, the C_α _RMSFs are more evenly distributed, and the mean value varies from 0.34 Å in the PIWI domain to 0.44 Å in the PAZ domain. The RMSF plots for both proteins are strongly peaked – Figure 4 shows that the peaks tend to reside in unstructured surface regions connecting secondary structures, with the exception of one peak which spans the α5 and α6 helices of the PAZ domain of *Pf*-Ago (residues E219 to Q237).

### Correlated motions

**Figure 5:**
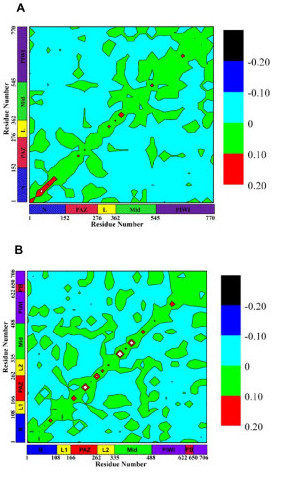
Pair correlations of atomic displacements calculated using Equation (3): (a) *Pf*-Ago, (b) *Aa*-Ago. Regions of high (*red*), medium (*green*), and low (*blue*) correlations are shown. Black represents large anti-correlation. The self-correlation (diagonal line) often has the highest value. The area and shape of off-diagonal islands are measures of the extent to which the motions of different domains are correlated.

To explore the correlated motions among the four domains in the proteins, we calculated the covariance matrices whose elements are defined by Equation (3). Figure 5 shows the contour plots of these matrices. In the contour plot, the diagonal line depicts the self-correlation of the each residue while the off-diagonal points depict the correlations between different residues. The isolated off-diagonal points are associated with higher-frequency and less-collective motions.

The numerous isolated off-diagonal regions of small-to-medium size (*i.e., ~25–50 residues) *in Figure 5 indicate that complex interactions exist among the four domains in *Aa*-Ago; in *Pf*-Ago, the isolated islands indicate simpler mutual interactions. This observation is consistent with the structural difference between *Aa*-Ago and *Pf*-Ago (Figure 1), i.e., *Aa*-Ago's 2+2 bi-lobal architecture and *Pf*-Ago's 1+3 architecture [11]. Thus, closer coupling is expected among the four domains in *Aa*-Ago than in *Pf*-Ago. For *Aa*-Ago, the strongest correlated motions occur between the PAZ and Mid domains. Although the PAZ domain has more contacts with the PIWI and N-terminal domains than the Mid domain, the long-range correlation between the PAZ and Mid domains is consistent with the collective torsional modes discussed below. Smaller correlated motions exist between the Mid and N domains and the Mid and PIWI domains. For *Pf*-Ago, the strongest coupled motions occur for the Mid and PIWI domains, followed by motions of the PAZ and N-terminal domains. A weaker interaction between the Mid and PAZ domains was also observed.

### Domain motions of *Pf*-Ago

#### Hinge motion opens and closes gate between N and PAZ domains in *Pf*-Ago

**Figure 6:**
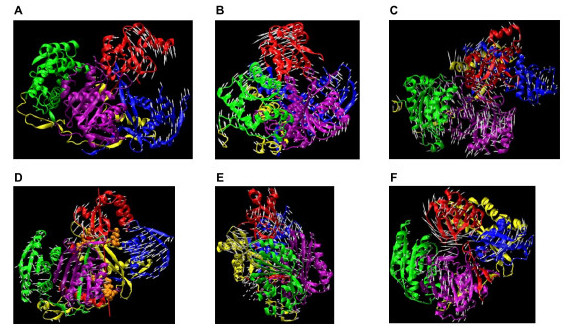
Argonaute normal modes. (a) Hinge motion (lowest frequency mode) between the N-domain and PAZ-domain of *Pf*-Ago. The hinge region, determined by Hingefind [24], is located between PAZ and N-terminal domains. (b) Torsional motion of *Pf*-Ago. In addition to the major torsion between the Mid-PIWI domains and the N-terminal domain, a torsion between PAZ domain with the rest of the protein was observed. (c) Breathing motion of *Pf-*Ago. Four domains synchronously move outwards/inwards to increase/reduce the volume of the protein. (d) Hinge motion (lowest frequency mode) between PAZ-containing lobe and PIWI-containing lobe in *Aa*-Ago. Red line, rotational axis of the hinge motion. Residues within 3.5 Å of the axis shown in van der Waals spheres. (e) Torsional motion (second lowest frequency mode) of *Aa-*Ago. A major torsion between the Mid-PIWI domains and the N-terminal domain was observed, while the PAZ domain moves associated with the Mid and PIWI domains. This motion changes the space spanned by the two lobes. (f) Breathing motion (the third lowest frequency mode) of *Aa-*Ago. PAZ domain, N-terminal domain and PIWI domain synchronously move outwards/inwards to increase/reduce the volume of the protein. Arrows added to C_α _atoms represent orientation and amplitude of the C_α_'s displacement in the mode.

The lowest-frequency (0.82 cm^-1^) mode in *Pf*-Ago is a hinge-motion between the N-terminal and PAZ domains. In this mode, the Mid and PIWI domains are relatively immobile (Figure 6a). A hinge-locating program was used to determine the hinge region [24]. The hinge region is located between the PAZ and N-terminal domains, including residues 118,119,122,123,151 of the N-terminal domain and residues 169–172 of the PAZ domain. In the "open" state, there is a large gap between the PAZ and N-terminal domains, which minimizes the interaction between the PAZ domain and the rest of protein. This motion separates the PAZ domain from the crescent base, making the nucleotide-binding pocket of the PAZ domain freely accessible for the binding of the 3' end of the siRNA template. The mode is consistent with instantaneous gating, where motion of the PAZ domain controls the entrance of siRNAs into the groove formed between the PAZ and PIWI domains. Cooperation with other modes (discussed below) is also necessary to accommodate and align the siRNA into the groove between PAZ and PIWI. In the "closed" state, the distance between PAZ and N-terminal domains is dramatically reduced, closing the gate.

#### Torsional motion of *Pf*-Ago

The second-lowest frequency mode of *Pf*-Ago involves a torsional motion, referred to as the *primary rotation*, where the N-terminal and PAZ domains rotate with respect to the Mid and PIWI domains with a frequency of 1.07 cm^-1 ^(Figure 6b). In this mode, the Mid and PIWI domains move rigidly while the N-terminal domain, together with the PAZ domain, adopts a reverse rotation. The rotation-axis has the direction from the connection portion between the Mid and PIWI domains to the linker between the N-terminal and PAZ domains. This direction roughly coincides with the extension of the groove (the blue shape from left to right with slight inclining) defined in in *Pf-Ago *structure (see Figure 4A in [10]). Interestingly, a second torsional mode (*secondary rotation*) involving the PAZ domain was observed with a different rotational axis. The secondary rotation has its axis directed from the PAZ domain to the PIWI domain, which is almost perpendicular to the primary rotation-axis. Of the four domains, the N-terminal domain has the smallest amplitude of motion in this mode.

#### Breathing motion of *Pf*-Ago

The next most interesting motion observed for *Pf*-Ago is a "breathing" mode characterized by the PIWI domain moving towards and away from the PAZ domain (Figure 6c). This mode has a higher frequency of 3.45 cm^-1 ^and ranked the 14^th ^lowest in the normal mode frequency spectrum, indicating that the corresponding conformational change may require ~11–12 times more energy than that associated with the above torsion and hinge modes. An important effect of the breathing mode is to change the volume of the cavity enclosed by the protein, in particular, the space between PIWI and PAZ domains. This mode also involves torsional motions between the N-terminal domain and the Mid domain, which appear to work in conjunction with the breathing motion to increase or decrease the volume of the cavity enclosed by the protein.

### Domain motions of *Aa*-Ago

#### Hinge motion between PAZ-containing lobe and PIWI-containing lobe in *Aa*-Ago

The lowest-frequency (0.97 cm^-1^) mode of *Aa*-Ago protein is a hinge motion with the Mid and PIWI domains (PIWI-containing lobe as defined in [11]) constituting one arm, and the PAZ and N-terminal domains (PAZ-containing lobe as define in [11]) constituting the other (Figure 6d). The rotational-axis was defined using VMD [25] and the hingefinder program [24]. Ten residues have Cα's within 3.5Å of the hinge rotation-axis. These residues are 186,187, 253–256 in the PAZ domain and residues 602,619,625,626 in PIWI domain and PIWI Box (Figure 6d).

#### Torsional motion of *Aa*-Ago

**Figure 7:**
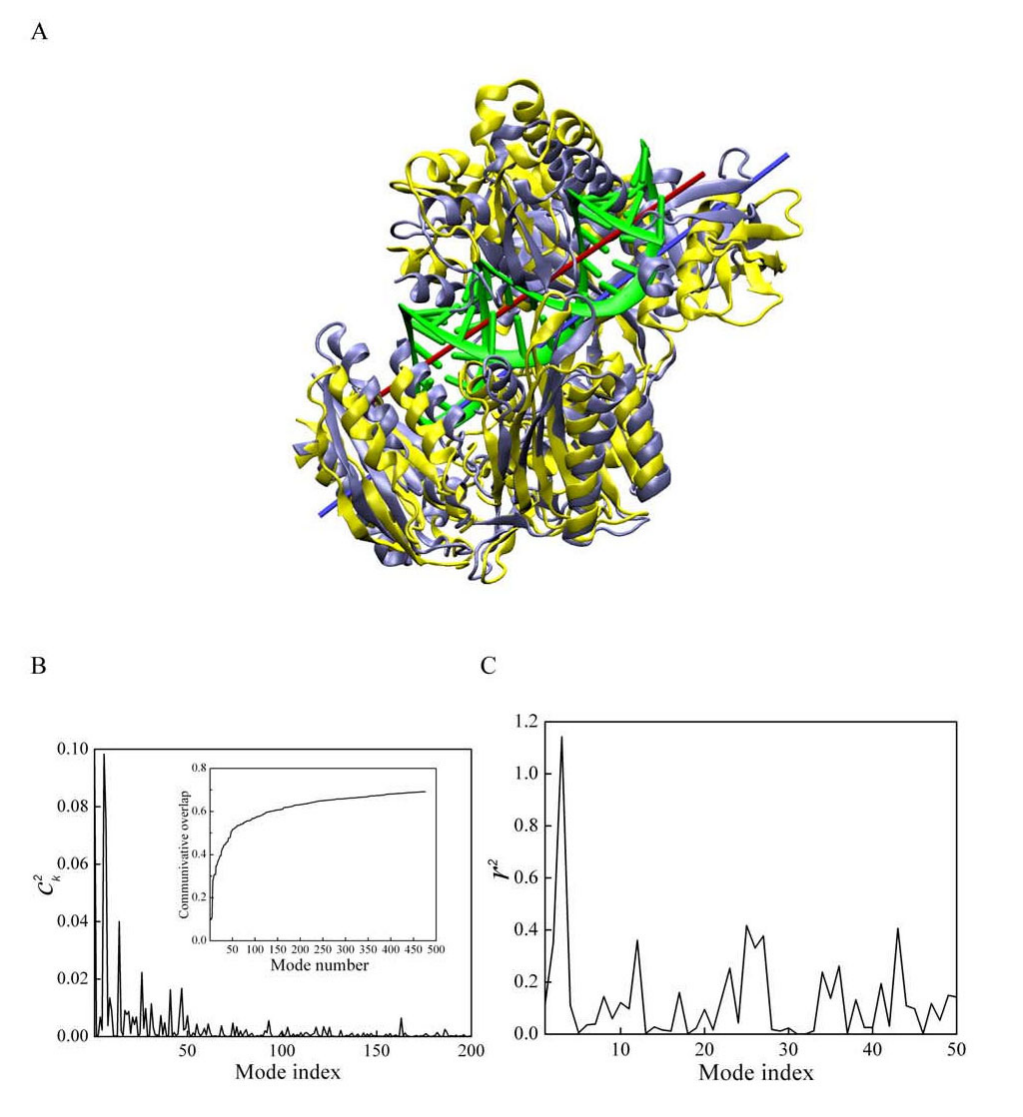
a) A model of Aa-Ago bound to guide DNA-mRNA. The hybrid duplex is colored in green, with the central red bar as its axis. As a comparison, the protein structure in its apo-form conformation is colored in light gray, and the one containing the hybrid duplex (model) is colored yellow. The yellow structure has an orientation similar to that in Figure 4b). b) The normal mode representation of the C_α _conformational changes of *Aa*-Ago in adopting DNA-mRNA duplex. The coefficient was calculated using Equation (5). The inset shows the cumulative contribution to the C_α _conformation change by each mode. c) The radius change of the imaginary cylinder, which encloses C_α _atoms located within 5 Å of the DNA-RNA duplex in panel a).

Similar to *Pf *-Ago, the second-lowest frequency mode (1.61 cm^-1^) in *Aa*-Ago involves a major torsion between the PIWI-containing lobe and the N-terminal domain (Figure 6e). The torsional axis extends from the N-terminal domain to the Mid domain (Figure 7a). In contrast with the torsional motion in *Pf*-Ago, the *Aa*-Ago PAZ domain does not involve a major rotation, but adopts an additional torsion motion with respect to the N-terminal domain. This secondary movement has a rotational axis extending from the PAZ domain to the N-terminal domain. Superposition of the two torsional modes produces a conformational change that allows the protein to change between "locked" and "unlocked" states. In the "locking" process, the PAZ domain rotates outwards, while the N-terminal domain and linker regions move towards the PIWI-containing lobe. In the "unlocking" process, the PAZ domain moves inwards and the N-terminal domain and the linker region moves away from the PIWI domain. Of the four domains in *Aa-*Ago, the PIWI domain has the smallest displacement.

#### Breathing motion of *Aa*-Ago

The third-lowest-frequency (1.72 cm^-1^) mode in *Aa*-Ago is a breathing-motion involving the PIWI-domain and the PAZ-containing lobes. Similar to *Pf*-Ago, the breathing mode in *Aa*-Ago can significantly change the volume inside the protein (Figure 6f). However compared with that of *Pf*-Ago, the *Aa*-Ago breathing mode has a negligible torsional component. The *Aa*-Ago breathing motion enlarges or reduces the distance between the PAZ and Mid domains, as well as the distance between the PIWI domain and PAZ-containing lobe. This motion opens and closes the channel formed by two structural gates (one formed by the PAZ and Mid domains, and the other by the N-terminal and PIWI domains) as well as the groove between the PAZ and PIWI domains. This single-mode gating motion is to be contrasted with the gating motions in *Pf*-Ago, which require a combination of the hinge and breathing modes.

### Normal modes required for *Aa*-Ago-siRNA structural model of Patel

In their paper [11], Yuan et al. presented a structural model (PDB code 2ADS) of the *Aa*-Ago protein complexed with a hybrid DNA/RNA duplex (Figure 7a). In this model, the RNA strand represents the target mRNA and the DNA strand represents the guide strand. A conformational change involving large motions in the PAZ domain and linker (132–167) was proposed for the insertion of the hybrid duplex into the *Aa*-Ago protein; the conformational change also involves smaller motions of the PIWI domain and N-terminal domain. To understand the Yuan et al. structural model in terms of normal modes, we used Equation (5) to calculate the overlap between the proposed conformational change and individual normal modes. Figure 7b shows the contribution of each mode to the conformational change proposed by Yuan et al. The largest contributions come from mode 1, 6 and 7. These modes involve 1) hinge motion between the PIWI-containing lobe and PAZ-containing lobe and 2) extensive PAZ domain torsion and Mid domain torsion. The torsional axis of one of the modes (produces torsion between the N-terminal and PIWI-containing domains) required for the accommodation by *Aa*-Ago of the hybrid duplex is represented by the blue bar in Figure 7a. This axis is approximately parallel to the duplex axis, yielding a projection along this axis of 0.91. The inset shows (Figure 7b) the cumulative contribution for the first 300 modes, showing that first 50 positive modes account for 70% of the C_α _displacement. Therefore, these low-frequency normal modes account for most of the proposed conformational change. On the other hand, 30% of the change is not accounted for by the lowest-frequency modes, indicating that significant additional structural rearrangements are involved in modeling the interaction between DNA-mRNA duplex and *Aa*-Ago protein. For comparison, we also performed calculations based on elastic network model [20,21], and found similar overlaps (0.75~0.8 with the first 50 modes).

The accommodation of the hybrid duplex by Argonaute requires a change in the enclosed volume. To determine the normal mode that produces the greatest change in volume that is relevant to duplex accommodation, for each normal mode, we calculated the change in volume of a cylinder defined by the C_α _atoms on Argonaute in the vicinity of the model duplex (Methods). Figure 7c shows the change in volume occupied by the duplex as a function of normal mode. The most effective mode for changing the duplex space is the breathing mode, with a few other slightly higher frequency modes (25 to 27) that are similar to the breathing mode, with more local motion at the interface between the two lobes.

## Discussion and conclusion

In the case of *Pf*-Ago, it was previously suggested that residues 317–320 might constitute a hinge region, enabling the PAZ domain to move towards and away from the crescent base [10]. In this hinge motion, the PAZ domain, stalk, and linker region (i.e., residues 103–310) would move as a unit. It was suggested that this motion could allow RISC loading and mRNA binding between the PAZ/N-terminal cleft and the N-terminal/PIWI cleft. The normal mode analysis lends support to this suggestion, with a low-frequency mode involving a hinge motion between the PAZ and N-terminal domains. In this mode, both the PAZ and N-terminal domains flex, resulting in hinges through the stalk, linker and connecting regions of the PAZ and N-terminal domains. This motion is also consistent with RISC loading and mRNA binding in the PAZ/N and N/PIWI clefts.

In the case of *Aa*-Ago, the normal mode calculations show more collective motion between the PAZ-containing and PIWI-containing lobes, entailing the entire Argonaute protein, with hinging in both the PAZ and PIWI domains, including the PIWI box, as suggested by Patel and co-workers [11]. Compared to *Pf*-Ago motions, *Aa*-Ago motions are more collective in nature, as reflected by the analysis of correlated motions in Figure 5.

Patel and co-workers described a 4-step model for RNAi target cleavage, which included (1) siRNA:mRNA duplex nucleation, where 2–8 base pairs are formed between the guide siRNA and the target mRNA; (2) siRNA:mRNA duplex propagation, where the duplex zippers to completion; (3) target cleavage, where the PIWI domain active site cleaves the phosphodiester bond between positions 10 and 11 of the mRNA target; and (4) mRNA release, where the cleaved mRNA dissociates from RISC [11]. The normal mode analysis enhances steps (1) and (2) of the model in several interesting ways.

Step (1), nucleation of the siRNA:mRNA duplex, is complex and poorly understood, involving many substeps. In one substep, Argonaute must first bind to the mRNA. However, because target mRNAs often contain secondary and tertiary structure, it is likely that binding of Argonaute to its target requires helicase activity to melt mRNA structure. The low-frequency hinge modes may aid in exposing the guide siRNA to the mRNA target. Binding entails the nucleation between the guide siRNA strand and the target mRNA. It is not clear exactly how many base pairs form during the nucleation step; however, it is thought that nucleation begins at the 5' end of the siRNA [26,27].

Once mRNA binding is achieved, another substep requires that Argonaute distinguish between correct and incorrect target sequences. This may occur by repeated binding and dissociation events, or by binding and scanning along the mRNA, in a manner similar to a polymerase, or even using mechanisms similar to those used by the ribosome, which detects the geometry of the codon-anticodon base pairs [28,29]. For example, Song *et al.*[10] suggested that *Pf*-Ago is able to sense the minor groove width of the dsRNA in a manner similar to RNase H.

Squeezing of the base pairs by hinge modes or breathing modes might increase the fidelity of target recognition, enhancing the stability of cognate base pairs and destabilizing non-cognate base pairs. As described above, the breathing mode is the most effective mode for increasing and decreasing the volume available for duplex accommodation. Furthermore, in the double-stranded RNA association mechanism, breathing motions might facilitate sampling of low-energy states of the siRNA:mRNA duplex inside the Argonaute cavity, enabling tighter binding between the mRNA strand and the single-stranded siRNA.

Similarly, the hinge motion between the PAZ-containing and PIWI-containing lobes (in the case of *Aa*-Ago), or the hinge motion between the PAZ and N-terminal domains (in the case of *Pf*-Ago), might facilitate target recognition by enhancing thermal sampling of high-affinity binding-site conformations. We note that the *Aa*-Ago N-domain is closely related to the catalytic domain of the replication initiator protein [30].

During Step (2), siRNA:mRNA duplex propagation, zippering of the full siRNA:mRNA target duplex occurs. If the target is cognate, the process culminates with the transfer of the 3'-end of the guide siRNA strand from its PAZ binding site to the N-terminal domain. Even though the present simulations were performed without the duplex, the primary torsion mode bears an interesting relationship to zippering activity: the axis of the N-terminal/PIWI domain torsional mode is approximately parallel to the model hybrid duplex axis. If significant in the presence of the duplex, this torsional motion might act to test the stability of siRNA:mRNA base pairs in a more precise fashion than the hinge or breathing modes. In addition, the hinge motion between the PAZ and N-terminal domains in the case of *Pf*-Ago might facilitate the transfer of the 3'-end of the guide siRNA from the PAZ domain to the N-terminal domain described in the Patel model.

Finally, we note that although target recognition might naively be expected to be similar during RNAi, RNA-dependent RNA polymerase (RDRP) activity, and transcription, there are dramatic differences in structure and dynamics among Argonaute, RDRPs, and RNA polymerases. These differences are almost certainly driven by differences in detailed functional requirements. For example, during RNAi, once the target is recognized, there is no need for translocation along the mRNA. During RDRP activity and transcription, however, the polymerase must move along the template one nucleotide at a time. In addition, the biological role of Argonaute in prokaryotes is currently unclear. As more is learned about the relations among the structure, dynamics, mechanisms, and biological functions of these proteins, it will be interesting to consider the implications for their evolutionary history, which will not only tell a rich and interesting story in its own right, but will also yield general insight into how complex and exquisitely scripted behaviors evolve in macromolecular systems.

## Methods

NMA was performed using the CHARMM simulation program [31]. First, the crystal structure was relaxed to a configuration ***x ***at a local minimum in the potential energy *U*(***x***), and the integrity of the minimized structure was confirmed [32]. To gain insight into large-scale motions, we excluded the crystalline environment. Next, the Hessian ∂^2^*U*/∂*x*_*i *_∂*x*_*j *_was calculated at the minimum; normal modes and frequencies were then obtained by solving an Eigenvalue problem. Finally, as in previous NMA studies of proteins [17-19], low-frequency modes were interpreted in light of the biological function of the protein complexes.

The atomic model of *Aa*-Ago was taken from the 2.9 Å X-ray structure (Protein Data Bank (PDB) entry 1YVU [11]). Hydrogen atoms were added to the protein using the HBUILD module of CHARMM [31] and waters in the x-ray structure were removed. The extended-atom model TOPH19 and the polar-hydrogen parameter set for proteins, PARAM19, were used for the energy minimization and normal mode analysis [33]. To avoid large charge-charge interactions, the distance-dependent dielectric constant ε = 2*r *was used to include screening effects due to bulk solvent. The non-bonded electrostatic interaction was shifted to zero at 7.5 Å and the corresponding cutoff distance for the non-bonded neighbor list update was 8 Å.

Prior to calculating the normal modes of the *Aa*-Ago protein, the structure was relaxed by energy minimization, including 200 steepest descent minimization steps followed by 200 steps of adapted basis Newton-Ralphson minimization (ABNR) [31] with constant harmonic constraints, used to remove excess strain. This was followed by 5000 steps of ABNR minimization with gradually decreasing harmonic constraints imposed. Finally, 2410 steps of ABNR minimization were carried out without constraints, using a gradient threshold of 0.01 Kcal/mol/Å^2 ^for termination of the minimization. As in previous normal-mode analyses of large systems [34,35], use of this gradient threshold resulted in a "minimized" structure that was similar to the crystal structure (the C_α _RMS deviation between the X-ray and minimized structure is 1.1 Å), but at the cost of yielding a significant number of negative-frequency modes in the normal-mode analysis. By comparison, we found that a smaller threshold of 0.001 Kcal/mol/Å^2 ^yielded no negative-frequency modes, but resulted in a 2.3 Å RMSD with respect to the crystal structure. As in previous studies [34,35] we chose to increase the fidelity of the "minimized" structure to the crystal structure at the cost of creating some negative modes in the normal-mode analysis.

To calculate the normal modes of the structure, the Hessian was calculated using the VIBRAN module of CHARMM and was diagonalized using the DIAG module. Eighteen modes with negative frequency were found; detailed examinations of these modes revealed that they only involve local side-chain displacements and not global conformational changes.

A similar protocol was used to calculate the normal modes of the *Pf*-Ago protein (PDB entry 1U04 [10]). The RMS deviation between the x-ray and the minimized structure is 1.3 Å for *Pf*-Ago. Six negative modes were found for *Pf*-Ago protein.

Analysis of two-point correlations of atomic fluctuations provides an overall picture of the collectivity of the internal motions within the protein structures. We analyzed two-point correlations using the covariance matrix of the atomic motions: the correlation between the motions of the *i*th atom and the *j*th atom is calculated using the normal modes as

$$\langle \Delta r_{i}\cdot \Delta r_{j}\rangle =\frac{k_{B}T}{\sqrt{m_{i}m_{j}}}\sum_{k}\frac{u_{ik}\cdot u_{jk}}{\lambda_{k}}$$

where **r**_*i *_is the displacement vector of the *i*th atom, *m*_*i *_is the mass of the atom, *k*_B _is the Boltzmann's constant, and *T *is the temperature. **u**_*ik *_is the (dimensionless) mass-weighted displacement vector of the *i*th atom within the *k*th eigenvector **u**_*k *_and λ_*k *_is the corresponding eigenvalue (units Kcal/g/Å^2^). The sum runs over positive modes. Here we are more interested in analyzing the overall correlations between residues, as opposed to individual atoms. Thus, the displacement **u**_*ik *_in Equation (1) was replaced by ${\tilde{u}}_{ik}$, the mass-weighted displacement of the center of mass of the *i*th residue in the *k*th eigenvector:

$${\tilde{u}}_{ik}=\frac{1}{\sqrt{M_{i}}}\sum_{n_{i}}m_{n_{i}}\frac{u_{n_{i}k}}{\sqrt{m_{n_{i}}}}=\frac{1}{\sqrt{M_{i}}}\sum_{n_{i}}\sqrt{m_{n_{i}}}u_{n_{i}k}$$

where *M*_*i *_is the total mass of the *i*th residue and *n*_*i *_is the index for all the atoms belonging to the *i*th residue. The correlation between the motions of residue *i *and *j *is thus defined by:

$$D_{ij}=\frac{k_{B}T}{\sqrt{M_{i}M_{j}}}\sum_{k}\frac{{\tilde{u}}_{ik}\cdot {\tilde{u}}_{jk}}{\omega_{k}^{2}}$$

The atomic RMSF was used to describe the flexibility of the proteins, which is the self-correlation and the diagonal term of the covariance matrix [36]. Following Equation (1),

$$\langle \Delta r_{i}^{2}\rangle =\frac{k_{B}T}{m_{i}}\sum_{k}\frac{{\left|u_{\text{ik}}\right|}^{\text{2}}}{\omega_{k}^{2}}$$

To assess the overlap between a specific conformational change and a normal mode, we denote the C_α _conformational change by Δ**x **= (Δ**x**_1_, Δ**x**_2_, ⋯, Δ**x**_*N*_), where Δ**x**_*i *_is the displacement of the *i*th C_α _atom. The overlap of a normal mode with this conformational change is evaluated as:

where $u_{k}^{\alpha }$ is the normalized vector consisting of the C_α _subspace of the *k*^th ^eigenvector [37,38]. From the decomposition, one may identify which normal modes contribute to specific conformation changes. (Note that because $u_{k}^{\alpha }$ is normalized, the sum of $c_{k}^{2}$ over all modes is greater than 1).

To investigate the implications of the normal mode analysis for the proposed conformational change upon

$$c_{k}^{2}=\frac{{\left(\Delta x\cdot u_{k}^{\alpha }\right)}^{2}}{{\left|\Delta x\right|}^{2}},$$

duplex formation, we used the hypothetical model of Patel and co-workers [11]. In particular, to study the variation of the volume occupied by DNA-RNA duplex with normal-mode motion, the model was used to locate C-alpha protein atoms within 5Å of the duplex. This set of atoms was then used to analyze deformations of the crystal structure in terms of duplex accommodation. The selected C-alpha atoms form the shape of cylinder, with its axis aligned with that of the duplex. We defined the radius *r *of the cylinder as the largest distance between any C-alpha atom and the axis – the radius for the unaltered crystal structure is denoted by *r*_0_. The volume of the cylinder, defined as *V *(*r*) = *π *× *z *× *r*^2^, where *z *is the length of the duplex, was used as a measure of the space available to accommodate the duplex. We then imposed a conformational change to the protein according to each normal-mode vector, using an amplitude corresponding to a RMSD of 0.05Å^2 ^per atom. (We also considered a frequency-dependent amplitude as in Eq. (1), which led to identical conclusions.) We found that *r *takes different values when imposing different normal-mode motions. The volume changes associated with normal-mode motions were calculated as Δ *V *= *V *(*r*) - *V *(*r*_0_).

## Authors' contributions

DM carried out the calculations, performed analysis, made the figures and wrote the paper. MEW helped formulate problem, helped perform analysis and helped write the paper. KYS formulated problem helped perform analysis, helped make figures, and helped write the paper.
